# Prosthodontic Rehabilitation of a Residual Post-surgical Cleft Defect

**DOI:** 10.7759/cureus.38364

**Published:** 2023-05-01

**Authors:** Shreya Colvenkar, Shaik Ameer, Afeefa Shaikh, Amreen Begum, Sanghavi Latha

**Affiliations:** 1 Department of Prosthodontics, MNR Dental College and Hospital, Sangareddy, IND; 2 Department of Oral Medicine and Radiology, Panineeya Institue of Dental Science and Research Center, Hyderabad, IND

**Keywords:** prosthodontic, cast partial, cleft palate, denture, prosthesis

## Abstract

Cleft lip/palate is a common birth defect globally, and the impact of this condition on the dental health of affected individuals can be profound. During intricate rehabilitation of cleft lip and palate patients, the final phase is achieved with definitive prosthodontic treatment. Prosthodontic rehabilitation is often necessary due to missing teeth and the alveolar ridge, malocclusion, residual defects, and the discrepancy between maxillary and mandibular arches. This article presents a case report of a young female patient with residual post-surgical cleft palatal defect having a mobile anterior segment with missing lateral incisors rehabilitated by a cast partial denture. The prosthesis utilized provided improvements in the patient’s speech and esthetics but at a low level of cost and ongoing maintenance.

## Introduction

Cleft lip and palate are extremely common birth defect that is found in about one in every 800 births and affects millions of children and families worldwide [1]. The cause of cleft lip and palate is complex as it involves both genetics and environmental factors such as hereditary elements, psychological stress, malnutrition, or radiation exposure during pregnancy [2-4].

Proper management of cleft lip and palate requires long-term commitment and a multidisciplinary team approach [5,6]. A cleft palate team is composed of a variety of specialists in dental, medical, and allied healthcare fields. This team works together to provide comprehensive care for patients with cleft lip/palate. The dental specialties include orthodontists, oral surgery, pediatric dentistry, and prosthodontics. Prosthodontists play an important role as members of this team. Missing permanent incisors, residual palatal defects, rudimentary teeth, missing alveolar ridge, and malocclusion are often seen in cleft lip and palate patients [7-11]. Each of these factors influences the type of treatment (fixed or removable) chosen during rehabilitation. Invasive treatment involves bone grafting together with implant-supported removable or fixed prostheses, whereas a conventional removable or fixed prosthesis is beneficial for patients who refuse surgical intervention [12-20]. There are several prosthetic rehabilitation methods available for patients, ranging from non-surgical removable or fixed prostheses to surgical implant-supported options. The type of treatment chosen is dependent on factors such as the extent of the defect, the number of teeth remaining, and the amount of bone support present. Patients with tissue deficiency, multiple fistulae, soft palate dysfunction, or uncoordinated nasopharyngeal sphincter action resulting in hypernasal speech, are particularly well-suited for removable partial dentures (RPDs).

RPDs are the treatment of choice for cleft lip and palate patients who have multiple missing teeth and fistulae, tissue deficiency, and excessive edentulous space that cannot be spanned by fixed prosthesis [15-17]. This article presents a case report of a young female patient with residual post-surgical cleft palatal defect having a mobile anterior segment with missing lateral incisors rehabilitated by a cast partial denture.

## Case presentation

A 25-year-old female patient visited the Department of Prosthodontics for prosthetic rehabilitation of bilateral missing lateral incisors after the completion of orthodontic treatment. The patient presented with a repaired bilateral cleft involving the hard palate and the lip. The cleft lip had been repaired when the patient was three months old. The patient also provided a history of two attempts of surgical closure of the cleft palate when she was aged 11 years, which were not followed by prosthodontic rehabilitation due to financial constraints.

**Figure 1:**
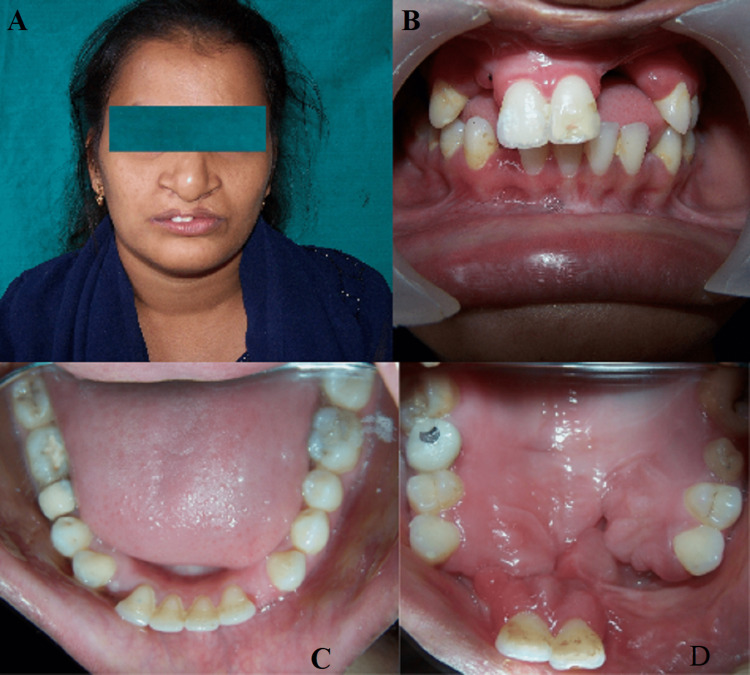
(A) Pre-treatment extraoral. (B) Intraoral frontal view. (C) Intraoral mandibular. (D) Intraoral maxillary.

On extraoral examination, the patient had a post-surgical scar on the left side of her lip. The upper lip was incomplete and raised on the affected side due to scar tissue contracture. Intraoral examination revealed post-surgical residual defects involving the anterior part of the hard palate, obliteration of sulcus in the maxillary central incisor region, missing lateral incisors, a mobile alveolar segment involving central incisors, absence of excess edentulous space around it, and diastema between the lateral incisor and canine in the mandible (Figures 1A-1D).

The option of a fixed partial denture (FPD) was rejected as there was no bone in the anterior region to support a FPD. Due to financial constraints, the patient did not agree to bone grafting and implants, or a FPD in the mandible. Instead, a cast partial denture was designed, which would provide good function and aesthetics by stabilizing the mobile anterior segment. Diagnostic impressions were made with irreversible hydrocolloids to formulate a treatment plan. A cast partial denture was planned in maxilla and RPD was planned for the lower jaw. The maxillary right premolar was restored with a metal-ceramic crown.

**Figure 2:**
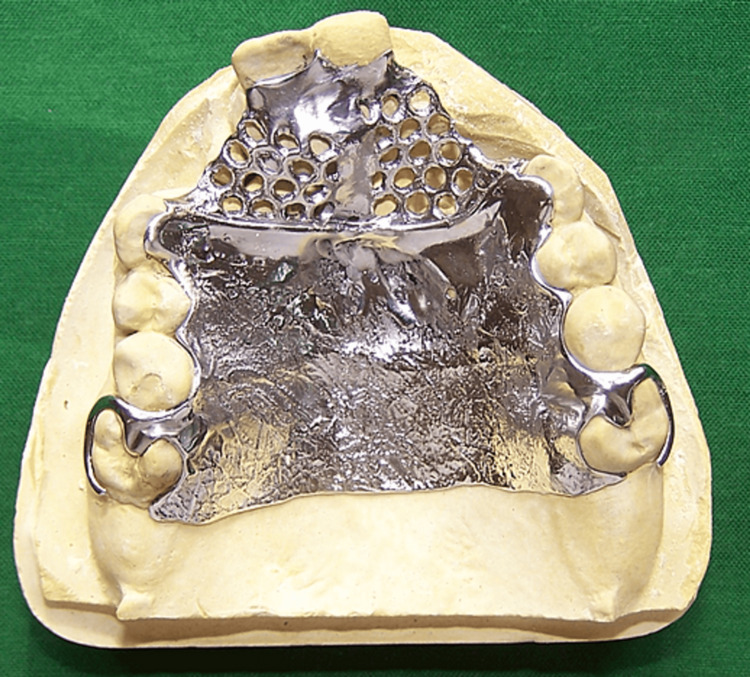
Cat partial denture framework

The diagnostic cast was surveyed to provide the design for the cast partial framework and the following design was decided upon. A complete palate major connector extending on palatal surfaces of teeth was used for better retention and stability. The mobile anterior alveolar segment involving central incisors was stabilized using a small linguoplate. Both sides of the bar were extended with mesh so that the defect could be sealed with acrylic followed by soft tissue conditioning with a tissue conditioner. Circumferential clasps were planned on the left and right first molars because of the presence of a distobuccal undercut. Indirect retention with canine rests was utilized to prevent rotation of the prosthesis. Rest seats were prepared on maxillary canines and first molars (Figure 2).

**Figure 3:**
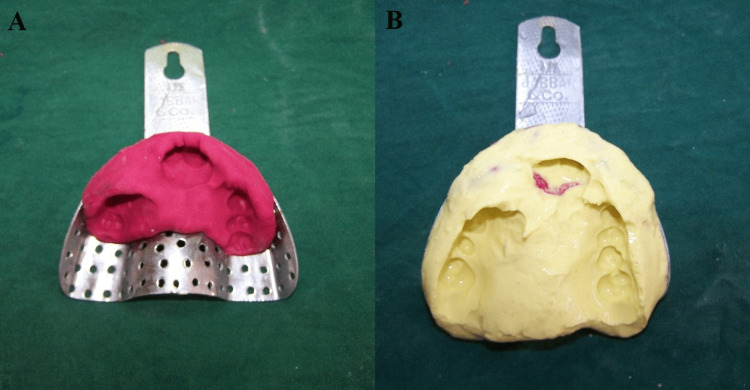
(A) Impression of the defect with impression compound. (B) Impression with alginate.

To record the full extent of residual post-surgical cleft defects, impressions were made with impression compound followed by alginate (Figures 3A, 3B).

**Figure 4:**
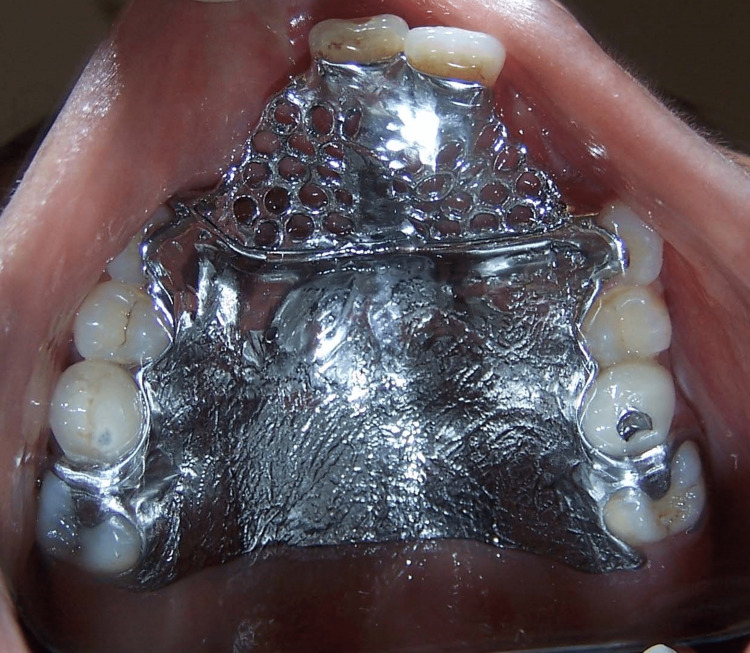
Cast partial framework try-in.

A fitting of the cast partial framework was performed to assess fit and comfort for the patient (Figure 4).

**Figure 5:**
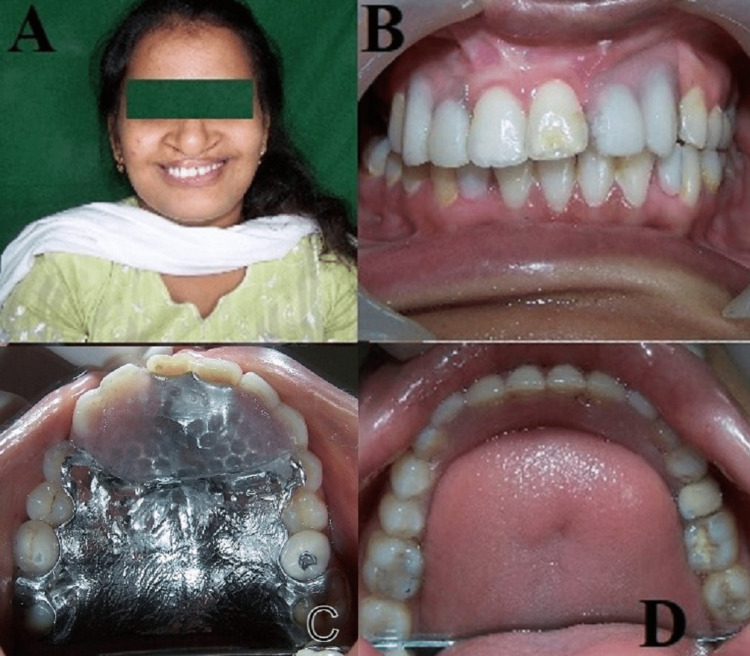
(A) Post-treatment extraoral view. (B) Intraoral frontal view. (C) Post-treatment maxillary. (D) Post-treatment mandible.

Jaw relations were then recorded. During try-in, a major hurdle was the presence of excess diastema between the central incisor and canine. Therefore, both the lateral incisor and canine were replaced instead of just the lateral incisor. An appropriate shade was selected which allowed the canine to be camouflaged as a premolar. The patient was then evaluated for esthetics. Speech was also assessed using tests such as perceptual and resonance frequency analyses. After the denture had been processed, it was tried in the patient’s mouth for a proper fit (Figures 5A-5D).

**Figure 6:**
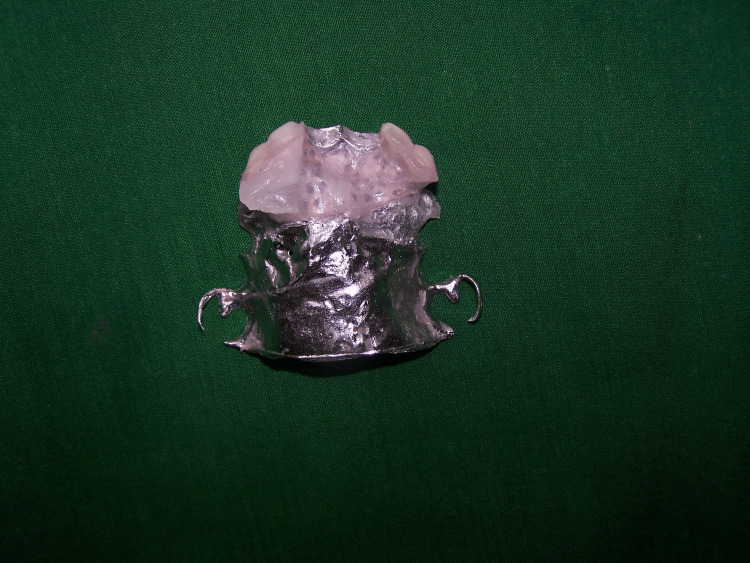
Removable partial denture with tissue conditioner.

The tissue side of the denture was reduced by 1 mm and tissue conditioner was applied in this area to provide a cushioning effect (Figure 6).

The patient was recalled every month for six months to check for any problems that may have developed. However, the patient was found to have adapted well to the prosthesis.

## Discussion

The prosthetic replacement of missing teeth is a crucial step in the rehabilitation of cleft lip and palate patients [12-20]. Several methods of prosthetic dental rehabilitation, outlined by Wegscheider et al. for CLP patients, include FPDs (such as prosthetic crowns and bridges or Maryland bridges), removable dentures (such as conventional cast partial overdentures and complete dentures), and precision prostheses (such as appliances with bars, splints, and telescopic retainers) [18]. The prosthetic treatment plan takes into consideration various factors such as defect form, speech and swallowing difficulties, dental abnormalities, cosmetic deformities, maxillary collapse, age, and financial status when deciding whether to use FPD or RPD [12].

Prosthetic rehabilitation of missing anterior teeth with large anterior ridge defects requires special consideration. Andrews Bridge is a fixed-removable prosthesis that provides complete closure of the defect, restoring speech and esthetics [16]. The only drawback is that the removable flange may cause plaque accumulation if not cleaned properly. In the present case, the patient was rehabilitated with a cast partial denture due to financial constraints.

During the replacement of missing anterior teeth, conventional FPD or implant-supported FPD is the most preferred option for the patient. But there should not be gross soft tissue deficiency in the alveolar ridge during replacement with FPD. Dental implants may improve the retention, stability, and occlusal function of prostheses in certain cleft palate cases [12-20]. For the successful placement of FPDs, it is essential that the alveolar ridge does not have any gross soft tissue deficiency. If present, then a grafting procedure is required to restore the function and aesthetics of the oral cavity, but it may not always be successful due to the quality and amount of bone required. In the present case, the patient was not interested in any surgical procedures for bone grafting and implant placement due to financial constraints.

To design an esthetic and functional prosthesis, the extent of malformations and resulting dysfunction must be considered. Since the defect was present in the anterior palate extending to the labial vestibule together with missing maxillary lateral incisors, it undoubtedly affected the patient's esthetics and speech. Due to the mobile anterior segment and residual post-surgical defect, treatment with an FPD was not suitable. A cast partial denture appeared as the treatment of choice for the patient as it would stabilize the anterior segment, thus enhancing functional loading and esthetics.

The patient also did not complain of any significant feeding issues but had speech problems. The patients' speech was challenging to understand after the RPD was removed. The palatal cover of the framework supported normal speech production and restore compensatory articulations. This design provided good retention and support from the hard palate. The thermal conductivity of the metal used for the denture helped the patient to sense temperature changes, thus improving functional acceptance by the patient. The cast partial denture also provided good support for the lips and yielded good esthetic results.

In edentulous anterior regions, vertical bone loss can cause severe hygiene problems and deficiencies in labial support. RPDs provide a viable solution to this problem by providing improved stability and support for the remaining teeth as well as offering better aesthetics. Furthermore, RPDs are an economical treatment option for those who cannot afford more complex treatment.

## Conclusions

Cleft lip and palate are one of the most common birth defects, affecting thousands of children worldwide. The foremost objective of rehabilitation of cleft lip and palate patients is to restore missing oral and facial structures. A removable prosthesis is the best option when hard and soft tissue deficiencies are extensive. This paper describes the successful prosthodontic rehabilitation of a post-surgical cleft defect with missing maxillary lateral incisors by fabrication of a cast partial denture. The cast partial denture provided improvements in the patient’s speech and esthetics but at a low level of cost and ongoing maintenance. In addition, RPD was a non-invasive alternative to more invasive procedures like dental implants, which may not be an option for cleft palate patients due to bone density issues.
